# Meeting Report: GBIF hackathon-workshop on Darwin Core and sample data (22-24 May 2013)

**DOI:** 10.4056/sigs.4898640

**Published:** 2014-01-20

**Authors:** John Wieczorek, Olaf Bánki, Stan Blum, John Deck, Markus Döring, Gabriele Dröge, Dag Endresen, Philip Goldstein, Patrick Leary, Leonard Krishtalka, Éamonn Ó Tuama, Robert J. Robbins, Tim Robertson, Pelin Yilmaz

**Affiliations:** 1 Museum of Vertebrate Zoology University of California Berkeley, CA USA; 2 Global Biodiversity Information Facility, GBIF Secretariat, Copenhagen, Denmark; 3 California Academy of Sciences, San Francisco, USA; 4 The University of California at Berkeley, Berkeley Natural History Museums, Berkeley, California, USA; 5 Botanic Garden & Botanical Museum Berlin-Dahlem, Freie Universität Berlin, Berlin, Germany; 6 GBIF-Norway, Natural History Museum, University of Oslo, Oslo, Norway; 7 University of Colorado, USA; 8 Marine Biological Laboratory, Woods Hole, USA; 9 Biodiversity Institute, University of Kansas, USA; 10 RCN4GSC Project, University of California at San Diego, La Jolla, CA, USA; 11 Microbial Genomics Group, Max Planck Institute for Marine Microbiology & Jacobs University Bremen, Germany

## Abstract

The workshop-hackathon was convened by the Global Biodiversity Information Facility (GBIF) at its secretariat in Copenhagen over 22-24 May 2013 with additional support from several projects (RCN4GSC, EAGER, VertNet, BiSciCol, GGBN, and Micro B3). It assembled a team of experts to address the challenge of adapting the Darwin Core standard for a wide variety of sample data. Topics addressed in the workshop included 1) a review of outstanding issues in the Darwin Core standard, 2) issues relating to publishing of biodiversity data through Darwin Core Archives, 3) use of Darwin Core Archives for publishing sample and monitoring data, 4) the case for modifying the Darwin Core Text Guide specification to support many-to-many relations, and 5) the generalization of the Darwin Core Archive to a “Biodiversity Data Archive”. A wide variety of use cases were assembled and discussed in order to inform further developments.

## Introduction

Darwin Core (DwC) is a glossary of terms commonly used in the biodiversity domain. It was originally conceived to facilitate the discovery, retrieval, and integration of information about modern biological specimens, their spatio-temporal occurrence, and their supporting evidence housed in collections (physical or digital). The Darwin Core standard ([1], [2]), ratified in 2009, is broader in scope. It aims to provide a stable, standard reference for sharing information on biological diversity, not just specimens, and not just modern. With the advent of tools for data publishing using Darwin Core, the standard has been adopted quickly and is now used to mobilize the majority of specimen and observational records within the biodiversity and collections communities.

As a glossary of terms, the Darwin Core provides stable semantic definitions with the goal of being maximally reusable in a variety of contexts. This means that Darwin Core may still be used in the same way it has historically been used, but may also serve as the basis for building more complex exchange formats while still ensuring interoperability through a common set of terms. Thus, the updated Darwin Core is no longer strictly bound to occurrence data, and, together with Dublin Core (on which its ideas are based), is used to encode data about organism names, taxonomies and species information and distributions.

The Darwin Core Archive (DwC-A) [3] is an exchange format based on Darwin Core and used in the GBIF Integrated Publishing Toolkit (IPT) [4]. The central idea of this archive is that its data files are logically arranged in a star schema [5], with one core data file surrounded by any number of ’extensions’. Each extension record (or ‘extension file row’) points to a record in the core file such that many extension records can exist for each single core record.

**Records in the core data file can be one of two types (or classes) of biodiversity data:**
Occurrence - the category of information pertaining to evidence of an occurrence in nature, in a collection, or in a dataset (specimen, observation, etc.);
Taxon - the category of information pertaining to taxonomic names, taxon name usages, or taxon concepts.

As examples, the extensions provide a means of serving multiple identifications for a specimen, multiple images of a specimen, or multiple common names for a taxon. This is now possible due to the broadening of scope of the Darwin Core and a redefinition of its structure into a reusable glossary of terms. Structurally, the Darwin Core Archive is a zipped archive consisting of a set of text files (for example, TSV or CSV) with a simple descriptor to inform others how the files are organized. The format is defined in the Darwin Core Text Guide [6].

## Purpose of the meeting

For historical reasons, Darwin Core and Darwin Core Archive have had a strong specimen- and taxon-oriented focus, with either physical specimens or taxonomic names forming the logical anchor points for much of the information represented in the archive. Now that molecular, and in particular genomic and metagenomic tools have become available for biodiversity research, there is a pressing need to add these kinds of data, and insights and inferences generated from these data, into information resources that deal with biodiversity and collections management data. Several efforts have been underway to facilitate the integration of genomic and metagenomic data with traditional biodiversity and collections information.

This report derives from a workshop that was organized to address some of these molecular, genomic, and metagenomic issues. The workshop was convened by GBIF with additional support from several projects: RCN4GSC, EAGER, VertNet, BiSciCol, EU BON, GGBN, and Micro B3 (see acknowledgements).

**The goals of the workshop were to:**
1. review the outstanding issues of the Darwin Core standard [1];
2. review issues arising from real-world publishing of biodiversity data through Darwin Core Archives;
3. explore mechanisms to publish sample and monitoring data using the existing Darwin Core Text Guide specification [6];
4. review the merits of modifying the Darwin Core Text Guide specification to support many-to-many relations, and assess the potential generalization of Darwin Core Archive to a “Biodiversity Data Archive”.

## Participants

The participants (see Participant List) were chosen for their technical knowledge of the various standards and/or knowledge of use cases relating to sampling protocols and sample data in various disciplines.

## Outputs

### Goal 1: Darwin Core outstanding issues

A summary was provided of the Darwin Core standard, its history, components, documentation, issue tracking, and governance. Specific issues relating to the remaining goals of the workshop were reviewed. Among these was the proposal ([7], [8]) to the biodiversity community to add a MaterialSample class and associated properties to the Darwin Core type vocabulary [9].

### Basis of record

The current Darwin Core Basis of Record type vocabulary terms associated with the occurrence class all describe artifacts of discrete organisms: PreservedSpecimen, FossilSpecimen, LivingSpecimen, HumanObservation, and MachineObservation. Since the use cases considered for this workshop involve looking at assemblages of organisms in various environments, plots, surveys, and sets of organisms processed as a unit for metagenomic samples, there was a need to consider terms that could adequately characterize these containers of diversity. Prior to this workshop, a proposal was made to the Darwin Core Standard for a MaterialSample Basis of Record type term, defined as:

“A resource describing the physical results of a sampling (or sub-sampling) event. In biological collections, the material sample is typically collected, and either preserved or destructively processed.”

Also discussed was the need for a Sequencing Basis of Record type term to handle the case where organisms are sequenced to derive the associated scientific name. Other possible Basis of Record type terms include Plot, Survey, and Object Aggregate. While discussed, no conclusion was reached on whether to implement these, or whether they can adequately be represented by MaterialSample.

### Habitat and ENVO

The Environment Ontology (ENVO) [10] provides a more granular way of referring to the environment in which an organism lives than is currently possible with the Darwin Core habitat term. In addition to “habitat” [11], ENVO provides three broad classifications for environment - biome, feature, and material. For example, in describing the environment inhabited by a particular individual bird, we would describe the material as “air” [12], the feature as “flood meadow” [13], and the biome as “flooded grassland biome” [14]. Microbial communities may be more significantly affected by their environmental material than a bird, as the microbe more directly interacts on this scale. The advantage of integrating Darwin Core with ENVO is that it provides a mechanism for integrating environmental descriptions for a broad range of species. Further, ENVO provides distinct URIs that can be used to denote the exact material, feature, or biome in question, making the content more semantically precise. Thus, it is recommended that the value of the Darwin Core habitat property be selected from the ENVO habitat class. For publishing using Darwin Core Archives, the ENVO label for the term should be used, e.g., “brackish water habitat” while, if publishing the data in RDF (e.g., using D2RQ [15]), the URI [16] should be used. It is also recommended that Darwin Core include three new properties (environmental material, environmental feature, and biome), the recommended vocabulary for which should be from the equivalent ENVO classes.

All use cases discussed in the workshop required the creation of a connection between individual organisms and some environmental context, whether that context was a physical medium such as a jar of water, survey, plot, another organism, or material associated with another organism. Handling these use cases using the Darwin Core standard or Darwin Core Archives necessitated the use of techniques to join organisms to other organisms, organisms to samples, or samples to samples. Handling these relationships suggests the use of globally unique instance identifiers, enabling integration not just within a Darwin Core Archive, but also across multiple archives and across domains. For example, a single material sample representing a collected instance of seawater may contain 1,000 distinct taxonomic occurrences of microbes that could be represented in the GBIF catalog, while the same sample could be characterized using genomic standards (MIxS) for representation in INSDC [17] or MG-RAST [18]. The standard concept obi:specimen [19] is generic enough to accommodate a wide variety of use cases (e.g., seawater, organism, soil). Instances of obi:specimens, represented by globally unique identifiers, can be shared among multiple standards, allowing inference of relationships between samples and their derivatives across multiple databases.

The essential features of instance-level identifiers are 1) resolution through HTTP or an HTTP proxy, 2) global uniqueness, and 3) persistence through time. Using globally unique identifiers as keys with Darwin Core Archives provoked concern amongst GBIF developers about the ability of data publishers to supply good identifiers consistently and to ensure that they are actually persistent. For example, in a preliminary, quick analysis, it was found that only 10 million out of approximately 100 million inspected records (DwC-A format) in GBIF had a dwc:occurrenceID complying with the essential features listed above. It should be noted, however, that most records had unique combinations of the triplet of dwc:institutionCode, dwc:collectionCode, and dwc:catalogNumber, which traditionally have been used to identify a record. This finding indicates that dwc:occurrenceID is either not properly understood or it is not used as the Darwin Core Archive authors expected.

The utilization of hierarchical identifier schemes was discussed as a possible solution to concerns about viable identifiers. These schemes allow one to assign, for instance, a single DOI or ARK group to designate all specimens in a particular dataset, using suffixes to denote locally specific identifiers. This strategy would allow one to reduce the number of registered identifiers greatly, since the locally unique suffixes could exist as unregistered identifiers within some larger scheme (see Biocode Commons Identifiers [20])

### Proposed new Darwin Core terms

Darwin Core provides several properties for describing sample data. These include dwc:eventID, dwc:samplingProtocol, dwc:samplingEffort, dwc:locationID, and dwc:individualCount. Two new terms were proposed to provide quantitative measures of the number of organisms in a sample. The new terms have been submitted to the DwC convener for ratification.

*abundanceAsPercent*- 100 times the number of individuals of a taxon found in a sample divided by the total number of individuals of all taxa in the sample.
*abundance*- the number of individuals of a taxon found in a sample. This is typically expressed as number per unit of area or volume. In the case of vegetation and colonial/encrusting species, percent cover can be used.

### Goal 2: Issues with real-world data publishing through Darwin Core Archives

Tools to produce and publish Darwin Core Archives (for example, IPT [4], Darwin Core Archive Assistant [21], and Darwin Core Archive Spreadsheet Processor [22]) have, by lowering the technical threshold of data publishing, made it easier to serve data, including “poor quality” data. While XML schemas can perform basic type validation (for example, integer, decimal, etc.), there is no automated mechanism for validating the delimited text used in the Darwin Core Archive format. GBIF has developed a Darwin Core Archive Validator [23], but there is still much that can be improved upon to catch errors and other data quality issues as early in the publication process as possible.

**Improvements might include:**
- sanitizing expected values of certain fields (for example, does a year look sensible);
- verifying referential integrity of related rows;
- verifying fields that should be using a controlled vocabulary are indeed doing so;
- verifying that the identifiers are present and being used as expected (e.g., not duplicated).

Recommendation for data improvements could be presented to the user as warnings (things that could be improved, or look suspicious) or errors (things that are demonstrably incorrect or inconsistent). Errors should be dealt with before being propagated further in the publishing process.

### Goal 3: Mechanisms to publish sample and monitoring data using Darwin Core Archives

The group developed and then analyzed several use cases covering various aspects of sampling and sampling procedures. The working goal was to demonstrate how Darwin Core Archives could be populated with example data for each of various use cases.

Darwin Core Archives are limited to using a star schema in which only a core record may be related to records in extensions. The core record can have relationships to as many extensions as desired and the relationship of the core to each extension can be one-to-many. Because of the limits on relationships between the core and extensions, it was assumed that two distinct types of core records would be needed to represent the use cases [Table 1].

**Table 1:** Two possible models, based on either an Occurrence core or a CollectingEvent core, for expressing sample use cases in Darwin Core Archives.

Use cases	Core type	Extension examples
● Specimen ● Botanical sampling event ● Bird spotting ● Oak branch with two lichens ● Whale tracking ● Camera trap ● Acoustic survey / telemetry ● Ocean acidification	**Occurrence** This core includes properties related to Event, Location, GeologicalContext, Identification, and Taxon.	● Images ● Identification history ● Sequences ● Related taxa (resource relationship) ● Measurement
● Vegetation plots ● Environmental sample ● Gut sample ● Plankton haul ● Trawl / subsample ● Towed diver survey ● Fisheries species abundance ● Checklist survey ● Fossil assemblage	**CollectingEvent** The core includes properties related to Event and Location	● Taxon Occurrence ● Measurements ● Images ● Survey geometry / course

The first model uses the GBIF Darwin Core Occurrence [26] as the core, always including the original collecting event and putting other events and sample information into extensions. The second model uses what would be a new core called “CollectingEvent” with other information such as occurrences, measurements, images, etc., in extensions.

Two template spreadsheets were created, one for a core file based on a Darwin Core Occurrence [24], and the second for a core file based on a Darwin Core Event [25].

Working groups were formed and assigned use cases. For each use case, groups were given the task of 1) selecting an appropriate model and making a copy of the appropriate template spreadsheet, and 2) populating the spreadsheet with metadata and data for the use case. Subsequently, the group reconvened to discuss the approaches used for each of the use cases and to determine what general recommendations could be made about the structure of Darwin Core Archives to represent various kinds of sampling.

Following is a list of the use cases considered. Where available, a reference to a link to a spreadsheet containing a worked example is provided next to the title of the use case. Spreadsheets contain separate sheets for the metadata, for the core file, and for any extensions required to fulfill the use case.

### Use Case - Seawater Environmental Metagenomic Sample** [****27****]**

The environmental metagenomic seawater use case is constructed from the broader goals of the Ocean Sampling Day project [28], which aims to catalogue microbial diversity across the world’s oceans via coordinated sampling on the two yearly solstices. Seawater samples are collected according to standardized protocols and metagenomic sequencing is performed, resulting in a list of microbial names assigned at the genus level. The context of the microbes depends on the sampling procedures used, including the depth at which the water was collected, filter size, and laboratory procedures used to derive the sequences. The resulting sequences are compared to known sequences with associated names to produce the list of resulting taxa and percentage abundance within the sample. The solution to this use case was constructed using an Occurrence Core with extensions for SamplingProcess, MeasurementsOrFacts, and ResourceRelationships.

### Use Case - Gut Microbiome Environmental Metagenomic Sample** [****29****]**

The use case is to describe procedure for and results of sampling the microbiome of the gut of an insect. The insect is associated with a specific time, place, and collecting protocol (e.g., caught with a net) where it was removed from its natural habitat. The environmental metagenomic sample is taken from the gut of the insect at a later time, referencing an anatomical location (gut), at a geographic location probably distinct from that of the collecting event (i.e., the laboratory where the sample was extracted from the insect), using protocols distinct from those used for collecting the insect itself. The challenge is to track the relationship of the microbes to the gut sample, the gut sample to the insect (including the process by which the gut sample was removed), and the insect to the collecting event. The solution to this use case was constructed using an Occurrence Core with an extension for ResourceRelationships.

### Use Case - Organism with Sub-sampling and Bulk Sampling - Moorea Biocode

In the Moorea Biocode [30] use case, the focus is on tracking a specimen, a subsample of that specimen that represents the organism itself (tissue sample), and a subsample of that sample that represents a new community (for example, gut flora). The specimen's gut is a new environment hosting a microbial community with many taxa. This community is sequenced as a whole (destructively sampled) with a set of 16S sequence reads describing the community biodiversity. These organisms may or may not have taxonomic names associated with them. There are identification processes associated with each type of subsample. The solution to this use case was not constructed in an example spreadsheet, but is similar in nature to a combination of the Tissue Sampling/Population Sampling and Gut Microbiome Environmental Metagenomic Sample use cases.

### Use Case - Botanical Sampling Event

A typical botanical sampling/collecting event is associated with several types of specimens and samples related to one collector's number. All of these samples and specimens (physical objects in a collection) result in unique identifiers (barcodes, catalogue numbers, or accession numbers). Preserved specimens are sometimes too large for one single herbarium sheet and will result in at least multiple sheets with distinct identifiers. Samples, specimens, and even collecting events can be documented with one or more images.

The solution to this use case was not constructed in an example spreadsheet. Nevertheless, the solution is straightforward. Include Occurrence Core records for each distinctly identified object and include identifiers for the individual organism (Darwin Core individualID) from which these objects were derived. Include image information in a media extension.

### Vegetation Surveys

Quantitative vegetation surveys can be permanent (linked to a fixed site) or carried out as one-off floristic surveys. The survey itself can either be based on a plot (a demarcated, structured area of land), or plot-less, for example, where distances from a sampling point (usually along a transect) are measured for a random sample of trees. The Vegetation Plot and Vegetation Relevé use cases below are examples of plot types, while the Point-centered Quarter is an example of a plot-less protocol.

### Use Case - Vegetation Plot [31]

A plot is a fixed demarcated square or rectangular area that is potentially subdivided into subplots, which may be divided into sub-subplots. For example, a typical forest plot might be a square of side length 100 meters (1 ha), containing 100 subplots of side length 10 meters. In these subplots, all individual trees above 10 cm DBH (diameter at breast height) are measured and identified. Depending on the nature of the forest plot, these subplots can, in turn, be divided into sub-subplots of side length 5 meter to capture all trees above 5 cm DBH. This nested design of subplots can go down to the level of subplots of side length 1 meter to capture the herb layer. The herb layer is estimated with percentage coverage. In the case where the forest plot is a permanent sample plot, all trees are issued with a tag referring, for example, to the plot number, the subplot, and the individual tree. Apart from the DBH, the point of measurement (POM) is noted. The POM is the exact point at which the diameter of the tree trunk is measured. This can differ from the diameter at breast height in the case of, for example, a buttress. The POM is painted so the diameter of the tree can be measured and compared over time. Usually a minimum of three plots are investigated per forest type. The solution to this use case was constructed using a CollectingEvent Core with extensions for Occurrences and MeasurementsOrFacts about the events.

### Use Case - Vegetation Relevés [32]

The Braun-Blanquet [33] vegetation plot, often called relevé [34] is typically used in phytosociological vegetation studies and commonly used in Europe. The plot, mostly a square, is chosen for its homogenous vegetation and must have a minimum size depending on the type of vegetation studied, for example, 100 m^2^ for forests, but only 1 m^2^ for grass communities. The Braun-Blanquet cover-abundance scale (and its derived, extended versions) is based on a combination of number of individuals and dominance, usually as a surface coverage in percent [35], [36], [37]. Typically, for each site, rich information such as soil and ground parameters, acidity, temperature, etc. are captured. The actual species occurrence information within the sample plot is rather quickly gathered, but can contain extra information about the species state, such as a phenological state ("flowering"), vitality ("weak"), or a reference to a captured specimen or a photo. The solution to this use case was constructed using an Occurrence Core with an extension for MeasurementsOrFacts about the occurrences.

### Use Case - Point-Centered Quarter

The Point-Centered Quarter method [37] is an example of a plot-less survey method, used, for example, for the accurate estimation of tree population densities in a forest. A transect is set up with a fixed number of random sample points along its length. For each quarter around the sampling point, the nearest tree is located and the following data are captured:

1. quarter number;
2. distance from sample point to center of tree trunk;
3. tree species (for example, from a checklist or a list of local tree names);
4. diameter at Breast Height (DBH) or Circumference at Chest Height (CCH).

Information may be collected in many forms, including specimen (for example, leaf, flower, fruit) vouchers, images, field notes, and measurements (for example, wood density) taken from individual trees.

The solution to this use case was not constructed in an example spreadsheet. However, the solution is the same as for most observation-only occurrences: 1) Include Occurrence Core records for each distinctly measured object and measurement information either in a MeasurementOrFact extension or in the core record within the Darwin Core term dynamicProperties, and 2) Include image information in a media extension.

### Use Case - Plankton Haul** [****38****]**

Marine plankton samples (for example, to measure zooplankton abundance [39]) are often associated with a particular station (location). The plankton haul follows a standard procedure using a variety of tools including water samplers and plankton nets [40]. A water sampler of known volume can be triggered to collect water (and microplankton) at a particular depth. A plankton net with particular mesh size, diameter ring, and flow meter is deployed horizontally for a fixed time/distance at a particular depth, or vertically through a depth range so that the volume of water filtered can be calculated. The trapped plankton are preserved and transferred to a bottle. Subsamples are taken from the bottle to identify species and make counts. Abundance is measured as #/liter (for microzooplankton) or #/cubic meter (all others). The solution to this use case was constructed using a CollectingEvent Core with an extension for Occurrences.

### Trawl subsample** [****41****]**

A trawl with sub-sampling (for example, in fisheries ecosystem research) involves capturing some detailed characteristics of a trawl sampling event, and capturing details about methods done subsequently to divide the total trawl into working units for investigation. The trawl, for example in the case of a towed net, represents a sample with geometric and other characteristics that can be captured in dedicated custom terms or in the Darwin Core terms samplingProtocol and samplingEffort. Geometric characteristics include the length of the trawl and the size and shape of the net. Other characteristics of the trawl may include species-specific or equipment-specific coefficients such as catchability. Further, the trawl sampling event may be associated with environmental conditions that are useful to record and correlate with subsequent analysis of contents.

The act of sub-sampling a trawl can mean dividing the entire contents of a trawl (for example, on the deck of the ship) into a smaller units, or subsamples, as desired for investigation. Other cases of sub-sampling can be more intricate, such as binning trawl contents by meaningful groups taxonomically or by size, sex or other characteristics. There is a requirement to enable identifiers for each subsample and to track each subsample back to the original sample. This connection helps maintain reference to the original trawl characteristics helpful for investigating the subsample. Often practitioners who use this kind of sub-sampling have such identifiers in place. It may not be necessary to recreate an entire data structure for both sample and subsample. It may be sufficient simply to "tag" the subsample with an identifier that ties it back to the original sample from which it came. In some cases, the characteristics of the subsample may be extrapolated back to the total sample, and in other cases, this function may not be required because the subsample itself may be a complete unit of study.

The solution to this use case was constructed using a CollectingEvent Core with extensions for Occurrences and MeasurementsOrFacts about the events.

### Towed-Diver Survey

A towed-diver survey involves visual identification, counting, and binning by a trained observer who is towed on an underwater sled through a portion of water where there may be fish to be investigated. These sampling events have a specific geometry based on the length of the tow and the relevant shape and dimensions of the visual space being scanned. This practice is intended to feed specific analyses of fish populations that can be input to repeatable quantitative biological models of fish stock. In addition to sample dimensions and the relevant biological dimensions required for binning (taxon, size, other observable characteristics), the data record may be required to record details about sampling conditions (such as bottom type, visibility, temperature) and even observational characteristics such as diver/observer identity.

The solution to this use case was not constructed in an example spreadsheet. However, the solution is the same as for most observation-only occurrences. Include Occurrence Core records for each distinctly observed target and put observed characteristics information either in a MeasurementOrFact extension or in the core record within the Darwin Core term dynamicProperties.

### Coastal Biodiversity Survey [**42**]

Rocky shore fauna are monitored twice yearly at fixed sites as part of a long-term ecological survey, based on the survey description provided by PISCO [43]. The survey area is typically 30 m wide along the shore (parallel to the water line). Starting at 0 m, 11 transects are established perpendicular to the line at 3 m intervals. The transects cover high, mid, and low shore regions. Three types of sample are taken:

1. Point contact sampling consists of at least 100 points along each transect surveyed - the first three species at a point are noted providing around 3,300 point samples.
2. Quadrat (plot) sampling consists of a 50 x 50 cm quadrat randomly placed in the high, mid and low zones of each transect; all mobile invertebrates are identified and counted. This results in 33 plots.
3. For certain rarer, key species, such as sea stars, a 2 m wide band centered on the transect line is sampled and the species name, number, and position along the transect are recorded.

The solution to this use case was constructed using a CollectingEvent Core with an extension for Occurrences.

### Checklist Survey** [****44****]**

In a typical checklist survey, a location is surveyed for the occurrence of species that are listed in a checklist. Provided the sampling/observation protocol is adequately described, this enables a measure of confidence in reporting the absence of a listed species at the survey location and time. The solution to this use case was constructed using a CollectingEvent Core with an extension for Occurrences.

### Oak Branch (or a rock) with Two Lichens** [****45****]**

An oak branch (or a rock) is collected, with two different lichens firmly attached. How can the information about the oak branch (rock) and the lichens be managed in a manner that preserves the hierarchical relationships among the branch (rock), the two attached lichens, and the lichens' composite fungi and algae? Note that the rock versus oak branch variation introduces the problem of an abiotic object as the root object in the hierarchy. The solution to this use case was constructed using an Occurrence Core with an extension for ResourceRelationships.

### Specimens from an Original and a Propagated Tree

As an example for this use case, a researcher takes a sample from a living tree in Borneo. Part of the sample is used to create preserved specimens as (one or more) herbarium sheets. Another part is rooted and used to create a living specimen in an arboretum. Several years later, another researcher takes a sample from the living specimen tree in the arboretum and, as before, part of the sample is used to create a preserved specimen while part is used to create a propagated living specimen in another location. This process could be repeated indefinitely. How can the information about these specimens be managed in a way to allow tracing the physical origin of all specimens (original or derived, preserved or living) back to the original collecting event in Borneo?

The solution to this use case was not constructed in an example spreadsheet. The recommendation is to include Occurrence Core records for each distinct event undergone by the identified object and include identifiers for the individual organism (Darwin Core individualID) in each occurrence record.

### Assemblage of one individual from fossil fragments collected at different times

As an example of this use case, in the early 1900s, Earl Douglass of the Carnegie Museum discovered a cache of fossil mammals in the Oligocene of Montana. In the 1990s, another researcher revisited the site and found partial bones that fit with fragments Douglass collected more than 80 years earlier. How can the "individual organism" (represented by the assembled fossil) be tied with the separately collected and catalogued fossil fragments that were acquired nearly a century apart? Normally we think of starting with an organism, then taking samples or subsamples from the organismal specimen, in which case we have the challenge of maintaining the connection with the original individual. Here, the subcomponents exist first, the individual emerges (or expands) later. To what extent does this reversal of direction of acquisition of parts to the whole affect (or not affect) its representation? The solution to this use case was not constructed in an example spreadsheet. The recommendation is to include Occurrence Core records for each distinctly cataloged fragment and “join” these with a common identifier for the individual organism (Darwin Core individualID) in each occurrence record.

### Genomic Analysis of Collected Scats

Predator scats are collected and subjected to DNA barcode sequencing to identify the prey species in the predator's diet. The existence of the DNA in the scat demonstrates the occurrence of at least one individual of a species in the predator’s diet, but further quantification cannot be done. In addition, the prey animal occurred where it was captured, not (necessarily) where the scat was deposited. If the home range of the predator is substantially different (quantitatively or qualitatively) than that of the prey, there will be some locational uncertainty between the actual location of the scat and the probable location where the prey was captured. For example, salmon DNA would be found in bear scats in the woods, but that does not mean that (living) salmon occur in the woods. How can such scat-derived evidence be represented in a Darwin Core Archive in a way that does not lead to material misrepresentation of the location of the DNA-identified prey species?

The solution to this use case was not constructed in an example spreadsheet. The recommendation is to include Occurrence Core records for each distinct taxon, each using the same Darwin Core Event and Location information. Relate the prey to the predator either through the Darwin Core term associatedOccurrences or through the ResourceRelationship extension. The distinction between the natural occurrence of the predator (“native”) and the dispersed occurrence of the prey could be captured in Darwin Core term establishmentMeans, though no recommended vocabulary currently exists for the dispersion described in this use case.

### Environment sample** [****46****]**

This use case was created to explore measurements of the environment or nature types without any simultaneous recording of an organism occurrence at the same time. This use case was not covered in group discussions and it is not clear if this use case is relevant for Darwin Core. Nevertheless, environment qualities and nature types provide an important element of biodiversity information and could provide improved understanding of species distributions. Environment information could furthermore often be recorded by non-biologists for other purposes not directly related to biodiversity. Formatting such environment information as a Darwin Core archive might improve the access for biologists to include these environment layers in predictive species distribution modeling. The solution to this use case was constructed using a CollectingEvent Core with extensions for MeasurementsOrFacts and Media about the events.

### Whale tracking** [****47****]**

A group of whales is observed by boat. Each time a whale surfaces, an observation is created with measurements about the surfacing event and approximate measurements of the whale’s physical characteristics. One of the whales is tagged with a tracking device, which periodically reports the geographic coordinates of the individual as well as measurements of the surrounding environment. In this example, the individualCount of the observations is set to one, which means the individualID can be used to identify one whale uniquely. The human and machine observations can be aggregated by individualID to get a complete account of one whale over time. The solution to this use case was constructed using an Occurrence Core with extensions for MeasurementsOrFacts and Media about the occurrences.

### Tissue sampling/Population sampling** [****48****]**

In this use case, 10 tissue samples (leaves) are collected for DNA analysis. A voucher specimen is also collected. All 11 objects belong to the same population, are taken on the same day at the same location, and putatively belong to the same taxon. One of the 10 tissue samples is taken from the same individual as the voucher. In addition, seeds from the original voucher are collected and cultivated in a botanical garden. Later, a voucher is also taken from the cultivated plant. The solution to this use case was constructed using a CollectingEvent Core with extensions for Occurrences and ResourceRelationships.

### Solutions without introducing an event core in Darwin Core Archives

During the review of the solutions for the uses cases, it became apparent that either model could be applied to every use case. The core and extensions bore a complementary relationship and between them could express all the required information. The core simply provided the central anchor in the star schema from which to join the additional information. Therefore, using the Occurrence core, well established in the GBIF network through uptake of the IPT, seemed more appropriate than inventing CollectingEvent as an additional core type.

### Extensions / List of new extensions

With the decision to use Occurrence as the preferred core for sample data, all sample processes will need to be included in extensions. It was decided that the location coordinates of the point where the parent sample was originally extracted from nature must be maintained with all taxonomic occurrence data. A sampling or sub-sampling process would be stored in a SamplingProcess extension with MaterialSample as the Basis of Record and joined to the Occurrence core with Occurrence identifiers. Taking into consideration earlier discussions between Darwin Core and the Global Genome Biodiversity Network (GGBN) [49] and DNA Bank Network [50] during the Biodiversity Information Standards (BIS TDWG) conference in 2012 in Beijing, the following extensions for sample data are proposed:

### ● SamplingProcess extension

### ● SampleProperties extension

### ● Preparation extension

### ● Preservation extension

### ● Amplification extension

One particular concern voiced at the workshop, but not discussed in detail, was that there already appears to be some confusion as to the perceived meaning of a Darwin Core Occurrence and its relation to a Darwin Core Event, and that the workshop recommendations for expressing sample data might further confuse matters. Brief discussions circulated around whether Occurrence was really understood incorrectly as a “Taxon Occurrence” or an “Organism Occurrence”, and if it did not have such a qualifier, how it was different from a simple Event, given event and occurrence are roughly synonymous in English. This discussion will continue in the Biodiversity Information Standards (BIS TDWG) community with a workshop to address the issue at the 2013 annual meeting in October.

## Goal 4: Generalization of Darwin Core Archive to “Biodiversity Data Archive”

The success of the Darwin Core Archive as the preferred format for publishing data to the GBIF network, coupled with the underlying text-based format being a formal part of the Darwin Core standard specification, has apparently led some to confuse the Darwin Core Archive with the Darwin Core vocabulary itself. The former, as a text file based data format, can potentially be used with vocabularies other than Darwin Core, and it has been suggested that the name “Darwin Core Archive” seems restrictive and might be changed to something more generic, for example, “Biodiversity Data Archive”, to reflect its general applicability. At the same time, the value, significance and uptake of the current Darwin Core Archive format was recognized, particularly with respect to the substantial tool and support ecosystem built around it. The following are issues relating to Darwin Core Archives that were discussed in the workshop.

### Desirable enhancements to current archives

Suggested enhancements include the addition of a multiValueDelimiter attribute to meta.xml fields, with a semicolon as the default value, and improved management of Darwin Core vocabulary extension definitions for the GBIF IPT. The delimiter enhancement is to overcome the diversity of verbatim original data, in which every conceivable character can be found in the content. The extension enhancement is to examine 1) their official status and relationship to the Darwin Core Text Guide specifications, 2) the central role of the GBIF Darwin Core Archive extension site [51], 3) the provision of lists of expected fields and data types including links to controlled vocabularies, 4) versioning, and 5) communal development of vocabularies.

### Darwin Core Archive name change

The Darwin Core standard itself does not use the term “Darwin Core Archive”, instead referring to the “Darwin Core Text Guide” [6]. Thus, the format used by what the community is calling Darwin Core Archive could be rebranded as Biodiversity Data Archive for broader use without affecting the current Darwin Core standard and with the possibility of continuing to use “Darwin Core Archive” in its current sense. The community would be free to have different flavors of archives, for example, “BDA-DwCA” for Darwin Core.

### Establish a separate archive standard

Establishing an archive standard separate from the Darwin Core (vocabulary) would allow both to evolve independently. A separate standard might more easily be enhanced with further constraint capabilities, typing, and a many-to-many relational model.

## Making Darwin Core Archive fully relational

A richer relational model is a requirement for networks such as Encyclopedia of Life (EOL) [52] and GGBN/DNA Bank Network. Google Data Set Publishing Language (DSPL) [53] was mentioned as an alternative, potential candidate system, but, while linked with the Schema.org [54] vocabulary system (of interest because of its use by big search engines such as Google, Yahoo, Bing, and Yandex), it was deemed unsuitable as it was out of direct community control.

**Any new archive system should:**
- be backwards compatible, so that old applications can still read the star schema
- provide, if possible, clearer semantics to support machine and human interpretation
- provide the option for multiple cores via multiple meta.xml files within one archive, enabling different views of an archive
- provide the option of adding table definitions with foreign keys
- provide archive versioning
- specify minimum metadata requirements for an archive, for example, based on a Dublin Core Application Profile [55].

The workshop agreed that a working session be proposed for the BIS TDWG 2013 conference to explore further the need for a General Biodiversity Archive format. This will be coordinated by EOL and GGBN/DNA Bank Network. A second working session is proposed for the BIS TDWG 2013 focusing on Darwin Core DNA and Tissue Data Standard for the Global Genome Biodiversity Network.

## Conclusions

An enhanced DwC standard is essential to support the data needs and interoperability challenges posed by global biodiversity networks such as the Group on Earth Observations Biodiversity Observation Network (GEO BON) [56], which will underpin the work of policy and decision makers, including the recently established Intergovernmental Panel on Biodiversity & Ecosystem Research (IPBES) [57].

Adopting DwC and the IPT as described in this report will help ensure that this popular standard becomes even more successful by enabling the encoding of a wide variety of sample-based data. This can be achieved by the addition of two new properties (abundance and abundanceAsPercent) to the DwC vocabulary and by using the IPT with the Occurrence core and appropriate extensions. Adoption of existing ENVO terms (environmental material, environmental feature, and biome) to expand on and standardize the current functionality afforded by dwc:habitat is also recommended.

Following the Darwin Core Namespace Policy [58] for making changes to the standard, all proposed changes arising from the workshop have been submitted to the TDWG Darwin Core Task Group with a view to ratification. Meanwhile, GBIF plans, over the coming year, to test the use of the enhanced DwC for publishing sample data to its network and thereafter promote its uptake. The GGBN plans to enable the use of Darwin Core Archive in parallel to ABCDDNA [59], [60] and BioCASe [61]. GGBN will test the proposed extensions over the coming year and will contribute to the documentation and review process.
